# Artificial Intelligence Self-Efficacy, Technostress, and Critical Thinking: Evidence from Peruvian University Students

**DOI:** 10.3390/ejihpe16070105

**Published:** 2026-07-21

**Authors:** Heyner Yuliano Marquez-Yauri, Sandra Lizzette León Luyo, Moises David Reyes-Perez, César Pol Arévalo-Aranda, Luisa Angélica Orejuela Guerrero, Carlos José Sandoval Reyes, Angelica María Minchola Vásquez, Marco Agustín Arbulú Ballesteros, Roger Ernesto Alarcón García

**Affiliations:** 1 Facultad de Ciencias Económicas, Escuela de Administración, Universidad Nacional de Trujillo, Trujillo 13011, Peru; saleon@unitru.edu.pe; 2 Facultad de Psicología, Escuela de Psicología, Universidad Nacional Mayor de San Marcos, Lima 15081, Peru; mreyesp@unmsm.edu.pe; 3 Departamento Académico de Ingeniería Metalúrgica, Escuela Profesional de Ingeniería Metalúrgica, Universidad Nacional de Trujillo, Trujillo 13011, Peru; carevalo@unitru.edu.pe; 4 Facultad de Ingeniería, Escuela Profesional de Ingeniería Industrial, Universidad Nacional de Trujillo, Trujillo 13011, Peru; lorejuela@unitru.edu.pe; 5 Institute for Research in Science and Technology, César Vallejo University, Campus Chepén, Trujillo 13001, Peru; cjsandovalr@ucvvirtual.edu.pe; 6 Escuela de Administración en Turismo y Hotelería, Facultad de Ciencias Empresariales, Universidad César Vallejo, Piura 20001, Peru; angelicamv@ucvvirtual.edu.pe; 7 Grupo de Investigación en Transformación Digital, Universidad Nacional Pedro Ruíz Gallo, Lambayeque 14013, Peru

**Keywords:** technostress, artificial intelligence self-efficacy, critical thinking, higher education, university students, PLS-SEM

## Abstract

The rapid integration of artificial intelligence (AI) into higher education has renewed concern that technology-related strain may erode students’ higher-order thinking. Drawing on a social–cognitive framework, this study tested whether AI self-efficacy and technostress predict critical thinking in 340 Peruvian undergraduates, who completed three validated scales (Yoon’s Critical Thinking Disposition Inventory; the brief General Self-Efficacy Scale for AI, GSE-6AI; and the RED-Technostress scale). Data were modeled with PLS-SEM, with significance from 5000 bootstrap resamples and an out-of-sample predictive assessment (PLSpredict, CVPAT). AI self-efficacy was a robust positive predictor of critical thinking (β = 0.42, *p* < 0.001), whereas technostress had no direct effect (β = −0.06, *p* = 0.50); AI self-efficacy was associated with higher technostress (β = 0.24, *p* < 0.001), but that path did not carry over to critical thinking (no mediation). A hypothesized moderation (AI self-efficacy mattering more at higher technostress) was not confirmed: it reached significance under the two-stage method but not under a stricter bootstrap, and the hypothesis was therefore not supported. The model explained a modest share of variance in critical thinking (R^2^ = 0.17) and was invariant across public and private universities. These findings reposition technostress as, at most, a distal correlate and identify confident, literate engagement with AI, rather than the absence of strain, as the more promising lever for cultivating critical thinking.

## 1. Introduction

The rapid incorporation of digital and artificial intelligence (AI) technologies into higher education has transformed how students access information, complete academic tasks, and regulate their own learning (Zawacki-Richter et al., 2019; Crompton & Burke, 2023). Since the public release of ChatGPT in November 2022, generative AI (GenAI) platforms have become the primary technological disruption in higher education, moving from the margins to the center of students’ academic routines, and a growing literature documents both their perceived benefits and the challenges they introduce (Chan & Hu, 2023; Strzelecki, 2024). As universities embed these technologies into curricula and communication platforms, the psychological demands placed on students have intensified accordingly, making the study of how digital strain interacts with cognition increasingly urgent.

One salient consequence of this intensification is technostress, the strain that arises when individuals cannot cope adaptively with information and communication technologies (ICT). First articulated in organizational research (Tarafdar et al., 2007; Ragu-Nathan et al., 2008) and later extended to broader populations (Salanova et al., 2013), technostress manifests through facets such as skepticism, fatigue, anxiety, inefficacy, and addiction. Among university students, technostress has been associated with diminished well-being, lower motivation, and weaker academic performance (Qi, 2019; X. Wang et al., 2021; Upadhyaya & Vrinda, 2021). Yet the evidence is not uniformly negative: some studies describe technology use as a “double-edged sword” whose consequences depend on how demands are appraised and managed (Qi, 2019), leaving open the question of how technostress relates to higher-order cognition.

Critical thinking: the ability to analyze, evaluate, and synthesize information to reach reasoned judgments, is widely regarded as a core graduate competency (Manalo et al., 2013; Shin et al., 2015). In contemporary higher education this competency must be exercised in technology-saturated, AI-rich environments, where students continuously appraise machine-generated information (Álvarez-Huerta et al., 2023; Meirbekov et al., 2022). It is therefore plausible that technology-related strain could compromise the cognitive resources critical thinking requires, yet the mechanisms that might protect critical thinking under digital strain remain poorly understood.

Two gaps motivate the present study. First, the direct link between technostress and critical thinking has seldom been examined empirically. Second, the proliferation of AI tools introduces a new and understudied personal resource: students’ self-efficacy for using AI. Building on Bandura’s (1977) social–cognitive account of self-efficacy and on decades of work on technology self-efficacy (Compeau & Higgins, 1995), researchers have only recently begun to operationalize AI self-efficacy specifically (Y.-Y. Wang & Chuang, 2024; Bewersdorff et al., 2025; Morales-García et al., 2024). Whether AI self-efficacy supports critical thinking, and whether technostress shapes that relationship remains unknown. Accordingly, this study tests (a) the direct effect of technostress on critical thinking, (b) the effect of AI self-efficacy on critical thinking, and (c) whether technostress moderates the AI self-efficacy–critical thinking relationship, in a sample of Peruvian undergraduates, a Latin American context in which technology integration is expanding rapidly and evidence remains scarce.

### 1.1. Theoretical Foundations and Hypotheses

The model described here draws on two traditions. Bandura’s (1977) social cognitive theory positions self-efficacy as a key component of motivation, perseverance, and self-regulation in complex cognitive activity. The challenge–hindrance stressor model (Cavanaugh et al., 2000; LePine et al., 2005) posits that not all demands are negative. It depends on the evaluation of those demands and the personal resources available to cope with them. Both perspectives suggest that critical thinking can be influenced by technostress and self-efficacy in AI, both separately and in combination.

#### 1.1.1. Technostress and Critical Thinking

Resource-based theories provide two complementary perspectives for explaining how technostress can have a detrimental effect on critical thinking. Cognitive load theory suggests that working memory has limited capacity. Additional demands due to poorly designed technology and extrinsic cognitive load compete with the processing required for analysis and evaluation (Paas et al., 2003; van Merriënboer & Sweller, 2005). The resource conservation model introduces a temporal aspect: students experience a burden and strain when technology fails to meet their needs and forces them to invest continuous effort to meet its demands. This burden and strain prevent students from engaging in high-demand computational tasks (Hobfoll, 1989; Hobfoll et al., 2018). In the context of the RED-Technostress model, fatigue, anxiety, and feelings of ineffectiveness reflect this dimension of exhaustion (Llorens et al., 2011; Salanova et al., 2013).

The empirical record consistently suggests a cost. College students’ well-being, motivation, and academic performance are negatively affected by technostress (Tarafdar et al., 2007; Ragu-Nathan et al., 2008; Qi, 2019; X. Wang et al., 2021; Upadhyaya & Vrinda, 2021), and negative effects have been observed in Latin America in Peruvian and Colombian samples (Alvarez-Risco et al., 2021; Andrade Navia et al., 2023). Critical thinking, however, is usually described as a stable, higher-order disposition that consolidates over years of schooling and may resist transient, day-to-day technological irritation (Hyytinen et al., 2018; Kocak et al., 2021), so a null direct effect is also plausible. Reading technostress in its conventional form as a hindrance stressor that drains cognitive resources, we nonetheless expected a negative direct effect:

**H1.**  *Technostress has a significant negative direct effect on critical thinking.*

#### 1.1.2. AI Self-Efficacy and Critical Thinking

Within Bandura’s (1977) sociocognitive theory, self-efficacy is the primary driver of cognition: students who are confident in their ability to succeed are more likely to set challenging goals, persevere, and engage in the metacognitive strategies essential for complex reasoning. Meta-analytic syntheses show that academic self-efficacy is most consistently related to performance and self-regulated learning (Honicke & Broadbent, 2016; Honicke et al., 2020). Furthermore, other studies have investigated the relationship between academic self-efficacy and critical thinking (Dehghani et al., 2011; Fahim & Nasrollahi-Mouziraji, 2013; Hyytinen et al., 2018).

It is reasonable to extend this reasoning to the field of AI for two reasons. First, self-efficacy appears to influence reasoning processes through creative and metacognitive channels that impact the quality of a judgment, rather than just the quantity of a judgment (Qiang et al., 2020; Fu et al., 2023). Second, as AI tools have become standard study aids, confidence in using such tools has been identified as a construct, distinct from general academic self-efficacy (Compeau & Higgins, 1995; Y.-Y. Wang & Chuang, 2024; Bewersdorff et al., 2025). Students who have confidence in their ability to effectively request and analyze AI output are more likely to challenge what the AI produces, rather than passively accepting it, and this is the emerging trend in AI-assisted learning (Meishar-Tal & Amzalag, 2026; Nasr et al., 2025).

AI self-efficacy is not computer self-efficacy under a new name. Computer self-efficacy concerns confidence in operating conventional, rule-based software whose behavior is predictable (Compeau & Higgins, 1995), and basic digital literacy covers the skills needed to locate and manage information online. Working with generative AI asks for something different: requests are phrased in natural language, output is probabilistic and can be plausibly wrong, and the user has to judge and iterate rather than execute. Confidence in that kind of exchange is what the GSE-6AI captures (Morales-García et al., 2024), which is why we treat AI self-efficacy as a distinct personal resource rather than a subtype of computer skill or digital literacy. We therefore hypothesized:

**H2.**  *AI self-efficacy has a significant positive effect on critical thinking.*

#### 1.1.3. The Moderating Role of Technostress

The challenge–hindrance framework regarding job demands suggests that demands have a different impact depending on the resources a person can mobilize to cope with them (Cavanaugh et al., 2000; LePine et al., 2005; Crawford et al., 2010). Resources that may be redundant when demands are low can be critical when demands are high. Resource conservation theory posits that when demands are high, resources are the most protective and the most likely to be mobilized (Hobfoll et al., 2018; Zeng & Cong, 2025).

**Figure 1:**
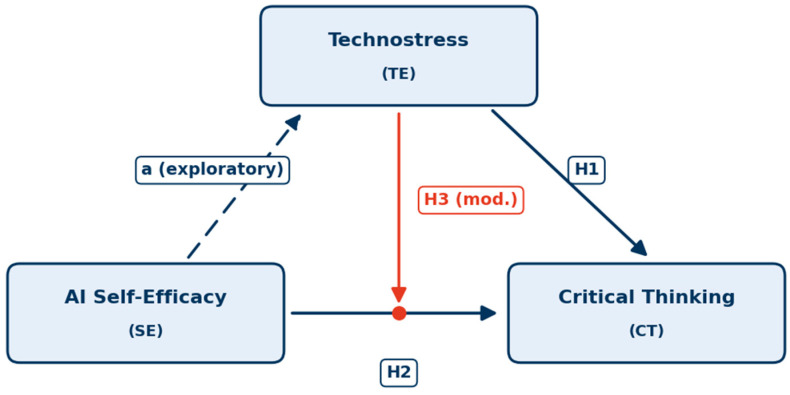
Conceptual model. H1: technostress → critical thinking; H2: AI self-efficacy → critical thinking; H3: technostress moderates the AI self-efficacy → critical thinking relationship. The dashed path a (AI self-efficacy → technostress) carries no hypothesis and is not part of the hypothesized structural model; it was retained only so the exploratory mediation reported in Section 3.6 could be estimated.

In the current case, when students experience low levels of technostress, they can maintain critical thinking through habitual routines, which may render differences in self-efficacy in AI insignificant. However, when technostress levels are high, and the information environment becomes more chaotic and exhausting, the ability to rely on AI to filter, verify, and synthesize information can improve the quality of a person’s thinking. Not every facet of the RED-Technostress scale should carry this interaction equally: we expected the strain facets, fatigue and anxiety, together with perceived inefficacy, to do the conditioning work, because these are the facets that drain the attention AI confidence helps to spare, whereas skepticism and addiction describe attitudes and usage habits rather than acute strain. Because technostress was modeled as a single composite, the test reported below pools the five facets, and facet-level moderation remains a task for future research. The relationship is expected to be more than additive, meaning that increased levels of technostress will not only accompany but also strengthen the relationship between self-efficacy in AI and critical thinking. Thus:

**H3.**  *Technostress moderates the AI self-efficacy–critical thinking relationship, such that the relationship is stronger at higher levels of technostress (Figure 1).*

## 2. Materials and Methods

### 2.1. Design and Participants

A quantitative, cross-sectional, correlational design was used. The sample comprised 340 undergraduate students from public and private universities in Peru, recruited through non-probability convenience sampling. The sample of 340 exceeded the minimum suggested by the inverse-square-root method for detecting the main structural paths (Kock & Hadaya, 2018), although statistical power for the interaction term was comparatively limited. Demographic characteristics appear in Table 1.

### 2.2. Instruments

Critical thinking was measured with 13 retained items from Yoon’s Critical Thinking Disposition Inventory (validated by Shin et al., 2015; 5-point scale). One item was removed prior to analysis after it was found to duplicate another item in the dataset. AI self-efficacy was measured with the six-item General Self-Efficacy Scale adapted for AI use (GSE-6AI; Morales-García et al., 2024; 5-point scale). Technostress was measured with 18 retained items of the RED-Technostress scale (Eidman & Basualdo Felleau, 2021; 7-point scale), covering skepticism, fatigue, anxiety, inefficacy, and addiction. Full item wording appears in Appendix A.

### 2.3. Data Analysis

Analyses used PLS-SEM (Hair et al., 2019), performed in SmartPLS 4 (version 4.1.1.8; SmartPLS GmbH, Monheim am Rhein, Germany). The measurement model was assessed for internal consistency (Cronbach’s α, ρA, composite reliability ρc), convergent validity (AVE), and discriminant validity (Fornell–Larcker and HTMT; Henseler et al., 2015). The structural model was evaluated through path coefficients, R^2^, effect sizes (f^2^), and out-of-sample predictive relevance (Q^2^predict and PLSpredict; Shmueli et al., 2019), complemented by the cross-validated predictive ability test (CVPAT; Liengaard et al., 2021). Moderation was tested with the two-stage approach, and the interaction was additionally re-estimated under a full bootstrap that re-estimates the measurement model in each resample. The structural model estimated for hypothesis testing contained the three hypothesized relations plus one auxiliary path from AI self-efficacy to technostress; that path carried no hypothesis and served only to permit the exploratory mediation test, so it is labeled exploratory wherever it appears. Significance and confidence intervals were obtained from 5000-resample bootstrapping; hypotheses were judged on bootstrap confidence intervals. Common method bias was examined with Harman’s single-factor test and the full-collinearity VIF (Kock, 2015). Finally, to test generalizability across institutional contexts, we ran a permutation-based multi-group analysis (MGA) comparing public and private university students, preceded by the measurement-invariance assessment of composite models (MICOM; Henseler et al., 2016). Finally, three robustness analyses added in revision, an item-purification trajectory, a higher-order re-specification of technostress, and an out-of-sample benchmark with regularized and nonlinear learners, are described and reported in Appendix B.

## 3. Results

Before the model was tested, the construct scores were inspected for distribution. Mean technostress was comparatively low (M = 2.19 on a 0–6 scale), whereas AI self-efficacy (M = 3.23) and critical thinking (M = 3.66) sat above the midpoint of their 1–5 scales (Table 2). Skewness and kurtosis stayed within plus or minus 2 for every indicator, which supports the use of PLS-SEM with bootstrap inference.

### 3.1. Measurement Model (Stage 1)

All constructs exceeded the 0.70 threshold for α, ρA, and ρc (Table 3). Standardized loadings ranged from 0.52 to 0.85; following Hair et al. (2019), items with loadings between 0.40 and 0.70 were retained to preserve content coverage and multidimensional structure of the validated source scales. AVE was 0.60 for AI self-efficacy and approximately 0.40 for both critical thinking and technostress. Although these two values fall below the 0.50 benchmark, their composite reliabilities remain well above 0.70 (0.90 and 0.92, respectively); Fornell and Larcker (1981) and Malhotra and Dash (2011) read a composite reliability above 0.60 as tolerable evidence of convergence when AVE falls below 0.50, although we recognize that composite reliability does not repair the item heterogeneity behind a low AVE. A modest AVE is, moreover, expected for the broad, multidimensional constructs assessed here (critical-thinking disposition and the five-facet RED-Technostress scale), whose heterogeneous content lowers average item communality. We nonetheless note the below-threshold AVE as a measurement limitation. Both re-specifications that a stricter reading of AVE invites, stepwise trimming of items with loadings under 0.60 and a model with the five RED facets as lower-order constructs beneath a global technostress factor, were run as robustness analyses and are reported in Appendix B: the facet-level model restores convergent validity at every level (facet AVEs 0.586–0.759; higher-order AVE 0.568) and the structural estimates barely move under either re-specification, so the below-threshold AVE affects the validity indices, not the conclusions. All indicator VIFs were below 3.3, indicating no indicator collinearity.

### 3.2. Discriminant Validity

Discriminant validity was supported. The square root of each construct’s AVE exceeded its inter-construct correlations (Fornell–Larcker), and all HTMT ratios were well below 0.85 (Table 4).

### 3.3. Common Method Bias and Model Fit

Harman’s single-factor test attributed 22.8% of variance to the first unrotated factor (<40%), and all full-collinearity VIFs were below 1.3 (CT = 1.20; SE = 1.28; TE = 1.07), indicating that common method bias was not a concern. Approximate model fit was mixed: the SRMR of 0.105 exceeds the conventional 0.10 cutoff and indicates limited exact fit, although the NFI of 0.952 is above the 0.90 benchmark; consistent with current guidance, model fit is de-emphasized in PLS-SEM and inference relies on the measurement and predictive assessments (Table 5; Hair et al., 2019). We note, though, that limited global fit occurs here alongside weak out-of-sample prediction (Section 3.4); the two shortfalls compound each other. Appendix B shows, though, that once the item set is re-specified the SRMR falls to 0.067–0.082 with unchanged structural conclusions, so the elevated value reflects item heterogeneity rather than structural misfit.

### 3.4. Explanatory and Predictive Power

AI self-efficacy and technostress explained 17.0% of the variance in critical thinking, and AI self-efficacy explained 5.7% of the variance in technostress (Table 6). Out-of-sample predictive relevance was limited: PLSpredict Q^2^predict was near zero for critical-thinking indicators (although PLS outperformed the linear-model benchmark on RMSE for all 13 indicators) and only marginally positive for technostress, and the CVPAT showed that the model did not surpass the indicator-average benchmark for critical thinking (Table 7). This pattern is consistent with technostress being, at most, weakly linked to critical thinking.

### 3.5. Structural Model and Hypothesis Testing (Stage 2)

**Figure 2:**
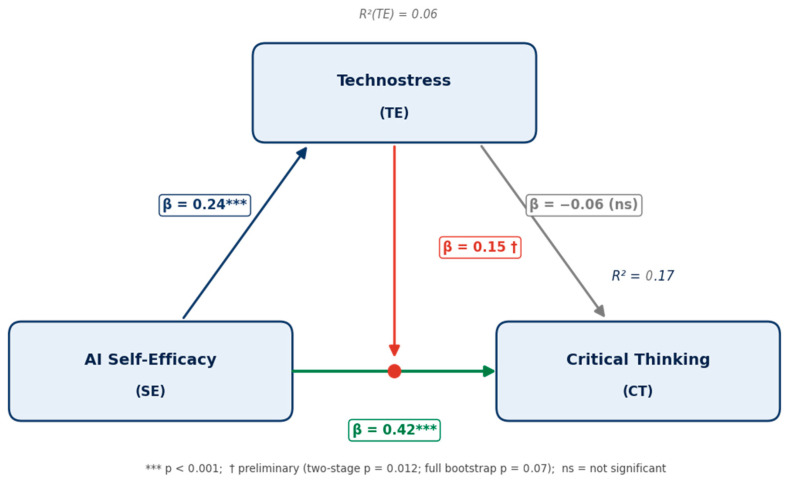
Estimated model with standardized coefficients. AI self-efficacy → critical thinking (H2) and AI self-efficacy → technostress (path a) was significant (*** *p* < 0.001); technostress → critical thinking (H1) was non-significant (ns); the moderation (†) was significant only under the two-stage approach.

AI self-efficacy had a robust positive effect on critical thinking (H2: β = 0.42, 95% CI [0.32, 0.53], *p* < 0.001, f^2^ = 0.20). Technostress had no direct effect (H1: β = −0.06, *p* = 0.50) and was therefore not supported. The AI self-efficacy → technostress path was positive and significant under the standard bootstrap reported in Table 8 (β = 0.24, 95% CI [0.122, 0.345], *p* < 0.001), but it did not remain robust once the measurement model was re-estimated in each resample (95% CI [−0.12, 0.36], *p* = 0.08), the same conservative check applied to the moderation in Section 3.6 and in Appendix B. Results appear in Table 8 and Figure 2.

### 3.6. Moderation (H3) and a Complementary Mediation Test

**Figure 3:**
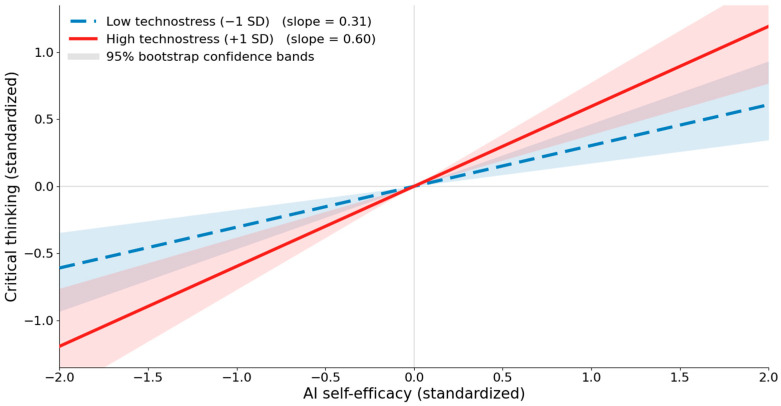
Technostress moderates the AI self-efficacy → critical thinking relationship (simple slopes). Shaded areas are 95% percentile confidence bands from the 5000-resample full bootstrap (Appendix B).

This section reports two complementary analyses of how technostress relates to the AI self-efficacy–critical-thinking link: the moderation hypothesized in H3 and an exploratory mediation test of whether technostress transmits AI self-efficacy’s effect on critical thinking. The moderation is reported as not supported: the interaction did not hold under full bootstrap re-estimation, and its term is underpowered (f^2^ = 0.03). The mediation is exploratory and was not hypothesized. The technostress × AI self-efficacy interaction on critical thinking was positive (β = 0.15, f^2^ = 0.031). Under the standard two-stage approach the interaction was significant (95% CI [0.024, 0.251], *p* = 0.012); however, under a full bootstrap that re-estimates the measurement model in each resample, it was only marginal (95% CI [−0.015, 0.264], *p* = 0.07). H3 is therefore not supported: an interaction that fails the stricter re-estimation cannot be read as evidence of moderation. Appendix B re-examines the term under both re-specified measurement models: it stays non-significant under the higher-order specification (*p* = 0.099) and reaches significance only when technostress narrows to its addiction facet (*p* = 0.024), a specification-dependence that supports rejecting H3 rather than reviving it. Simple-slope analysis (Table 9, Figure 3) indicated that the AI self-efficacy → critical thinking slope rose from 0.31 at low technostress (−1 SD) to 0.60 at high technostress (+1 SD); given the non-significant interaction under the full bootstrap, these slopes are descriptive only and carry no inferential weight.

As a complementary, exploratory analysis (not a formal hypothesis), the indirect path from AI self-efficacy to critical thinking through technostress was also examined (Table 10). The component paths were uneven: AI self-efficacy predicted technostress significantly (a = 0.24, *p* < 0.001), but technostress did not predict critical thinking (b = −0.06, ns). The resulting specific indirect effect was negligible and non-significant (a × b = −0.014, 95% CI [−0.039, 0.011]), whereas the direct effect of AI self-efficacy on critical thinking was strong (c′ = 0.42, *p* < 0.001). Following the decision rules of Zhao et al. (2010) and the PLS mediation guidance of Nitzl et al. (2016), this configuration corresponds to a direct-only non-mediation pattern (AI self-efficacy relates to critical thinking directly, not through technostress); the variance-accounted-for index is not interpretable here because the direct and indirect effects carry opposite signs. The exploratory mediation therefore received no empirical support. Table 11 summarizes the outcome of all hypothesis tests.

### 3.7. Multi-Group Analysis: Public Versus Private Universities

To assess whether the findings generalize across institutional contexts, we compared public (*n* = 213) and private (*n* = 127) university students. Measurement invariance was first established with the MICOM procedure (Henseler et al., 2016): configural invariance held by design, compositional invariance was supported for all three constructs (c = 0.88–0.99, each above the 5% permutation threshold), and the composites exhibited equal means and variances across groups (all *p* > 0.10), indicating full measurement invariance. With invariance established, a permutation-based multi-group analysis revealed no significant difference in any structural path between the two groups (Table 12): AI self-efficacy → critical thinking (β = 0.40 vs. 0.48, *p* = 0.43), technostress → critical thinking (β = −0.05 vs. 0.02, *p* = 0.74), AI self-efficacy → technostress (β = 0.28 vs. 0.32, *p* = 0.72), and the technostress × AI self-efficacy interaction (β = 0.20 vs. 0.02, *p* = 0.20). The model therefore holds equivalently in public and private universities.

## 4. Discussion

This study sets out to clarify whether technostress impairs critical thinking among university students and whether AI self-efficacy shapes that outcome. Three findings stand out. First, AI self-efficacy was a robust positive predictor of critical thinking. Second, technostress exerted no direct effect on critical thinking. Third, the hypothesized interaction, under which AI self-efficacy would matter more at higher levels of technostress, was not supported once the interaction term was subjected to full bootstrap re-estimation. Together, these results reframe a widespread concern: in AI-mediated higher education, the strain that technology imposes may matter less for higher-order cognition than the confidence with which students mobilize technological resources (Crompton & Burke, 2023; Chan & Hu, 2023; Nasr et al., 2025).

### 4.1. AI Self-Efficacy as a Driver of Critical Thinking

The robust effect of AI self-efficacy on critical thinking (β = 0.42) is best understood through Bandura’s (1977) social–cognitive theory, in which efficacy beliefs govern the effort, persistence, and metacognitive self-regulation that complex reasoning demands. Meta-analytic evidence establishes academic self-efficacy as one of the most consistent correlates of academic performance and self-regulated learning (Honicke & Broadbent, 2016; Honicke et al., 2020), and a parallel literature ties self-efficacy specifically to critical-thinking dispositions and skills (Dehghani et al., 2011; Fahim & Nasrollahi-Mouziraji, 2013; Hyytinen et al., 2018; Qiang et al., 2020; Fu et al., 2023). Our results extend this evidence to the AI domain: students who feel capable of deploying AI tools to manage academic tasks appear better able to interrogate, evaluate, and integrate machine-generated information, the core acts of critical thinking. This interpretation converges with emerging work showing that academic and AI-related self-efficacy accompany stronger critical thinking and creativity in AI-supported settings (Meishar-Tal & Amzalag, 2026; Nasr et al., 2025), and with the broader argument that confident, literate engagement with AI, rather than mere exposure, is what benefits learning (Y.-Y. Wang & Chuang, 2024; Bewersdorff et al., 2025; Carolus et al., 2023).

### 4.2. The Null Direct Effect of Technostress

Contrary to H1, technostress showed no direct effect on critical thinking. Although resource-based accounts, namely cognitive load theory (Paas et al., 2003; van Merriënboer & Sweller, 2005) and the conservation of resources model (Hobfoll, 1989; Hobfoll et al., 2018), predict that technological strain should deplete the cognitive resources reasoning requires, several explanations may reconcile theory with this null result. First, critical thinking is a relatively stable disposition rather than a momentary performance, and dispositions are less sensitive to transient strain than state-level outcomes such as fatigue or anxiety (Hyytinen et al., 2018; Kocak et al., 2021). Second, consistent with the “double-edged sword” view of student technology use (Qi, 2019; X. Wang et al., 2021), the effects of technostress may be appraisal-dependent: when demands are read as challenges rather than hindrances, they need not impair cognition (Cavanaugh et al., 2000; LePine et al., 2005; Crawford et al., 2010). Third, prior Latin American evidence indicates that technostress relates to academic outcomes indirectly (through exhaustion, cognitive load, or self-efficacy) rather than directly (Alvarez-Risco et al., 2021; Andrade Navia et al., 2023; Ma, 2025); yet the indirect path we tested was itself not robust, suggesting that any such mechanism is weak in this sample. Formally classified, the pattern is a non-mediation under the Zhao et al. (2010) typology (Nitzl et al., 2016): the exploratory route from AI self-efficacy through technostress to critical thinking does not hold, indicating that AI self-efficacy relates to critical thinking directly rather than through technostress, and that technostress is, at most, a distal correlate of critical thinking. The remaining association ran counter to the usual concern: students more confident with AI reported somewhat higher technostress (β = 0.24; significant under the standard bootstrap, though not under full measurement re-estimation, Section 3.5), plausibly because greater AI self-efficacy goes hand in hand with heavier, more sustained use of these tools. That strain did not carry through to critical thinking, so technostress appears here as a companion of intensive AI engagement rather than a brake on higher-order cognition.

### 4.3. The Hypothesized Conditional Role of Technostress (Not Supported)

Research on the interaction between technostress and AI self-efficacy is still at an early stage. From the challenge–hindrance/resource-demand perspectives, an individual’s assessment of their personal resources increases with the level of demand faced (Cavanaugh et al., 2000; LePine et al., 2005; Crawford et al., 2010; Zeng & Cong, 2025). Students facing low levels of technostress engage in critical thinking and perform routine activities, making differences in AI self-efficacy of little importance. However, when facing high levels of technostress, the ability to use AI with confidence becomes critical, and the slope of self-efficacy nearly doubles (0.31 to 0.60). Conservation of Resources (COR) theory explains that resources are more valuable and necessary when the situation is more demanding (Hobfoll et al., 2018). The interaction was significant under the two-stage approach but only marginal under the stricter bootstrap, a pattern typical of small interaction effects (f^2^ = 0.03) evaluated under conservative inference. The close, rapid, and widespread adoption of generative AI technologies by Peruvian university students suggests that moderating effects may be context- and cohort-specific. Consequently, we reject H3: the pattern runs in the theorized direction, but an estimate that fails conservative inference is not a finding, and any conditioning role of technostress is left to larger, longitudinal designs with the power to detect small interactions. Appendix B adds that the estimate is specification-dependent, emerging only when technostress is narrowed to its addiction facet, which strengthens the case for treating it as a targeted hypothesis rather than a finding.

### 4.4. Predictive Performance and Transparency

The predictive battery is reported at the indicator level. For the critical-thinking indicators, out-of-sample relevance was marginal (Q^2^predict close to zero), and the CVPAT showed the model performing no better than the indicator-mean benchmark. This is unsurprising: although AI self-efficacy is a strong in-sample predictor, critical thinking is a broad disposition shaped by many determinants beyond those modeled here, so the model explains only a modest share of its variance (R^2^ = 0.17) and offers little prediction beyond the mean (Kocak et al., 2021; Van et al., 2022). Reporting this limited predictive performance transparently, rather than overstating it, is itself a contribution in the context of PLS-SEM fit and prediction (Hair et al., 2019; Shmueli et al., 2019). Two further points follow. First, statistical significance should not be confused with practical significance: the effect of AI self-efficacy is reliable and of medium size (f^2^ = 0.20), but the model leaves 83% of the variance in critical thinking unexplained, so significance alone is a thin warrant for strong practical claims. Second, where out-of-sample prediction becomes the goal, a sensible route is to extract latent variable scores from an optimized measurement model and feed them to a regularized or nonlinear learner, such as ridge regression or support vector machines, benchmarked against the indicator mean. Appendix B reports exactly this benchmark: under ten repetitions of 10-fold cross-validation, ridge regression (out-of-sample R^2^ = 0.154) and support vector regression (0.131) do not outperform ordinary least squares (0.156), and none approaches the in-sample R^2^ of 0.17, which locates the modest predictive ceiling in the constructs measured rather than in the estimator.

### 4.5. Theoretical and Practical Implications

Theoretically, the study develops social–cognitive theory within the context of AI by considering self-efficacy in AI as a proximal predictor of advanced cognition, while reconceptualizing the relationship between personal resources and technostress as a boundary condition where technostress is not perceived as a direct threat (Hobfoll et al., 2018; Crawford et al., 2010). Practically, the results imply that reducing technostress alone should not be the primary focus of interventions; rather, positive and effective student engagement with AI should be promoted through AI literacy education and the constructive and evaluative application of AI (Crompton & Burke, 2023; Chan & Hu, 2023; Nasr et al., 2025). For Latin American universities, which are in the process of expanding their digital infrastructures, incorporating elements of trust in AI and critical evaluation into flexible digital literacy training may be more effective in safeguarding critical thinking than interventions aimed at stress response, particularly for students operating in high-tech environments (Alvarez-Risco et al., 2021; Andrade Navia et al., 2023). Multi-group analysis further showed the model to be invariant, both in measurement and in structure, across public and private universities, indicating that these implications are not confined to a single institutional context.

Several limitations qualify these conclusions. The cross-sectional design precludes causal inference; longitudinal and experimental work is needed to establish temporal order, especially for the proposed moderation. Non-probability convenience sampling within a single country limits generalizability, and reliance on self-report invites common-method concerns, although Harman’s test and full-collinearity VIFs indicated that such bias was not substantial (Kock, 2015). Voluntary participation adds a selection risk of its own: students who feel at ease with AI, or who are unusually bothered by technology, are the most likely to answer a survey on the topic, so both tails of the technostress distribution may be overrepresented and the estimates biased in either direction. Probability or quota sampling across institutions and disciplines should precede any claim of generalizability. Relatedly, the average variance extracted fell below the 0.50 benchmark for critical thinking and technostress; rather than trimming items to inflate this index (which would have collapsed the technostress measure onto a single facet and narrowed the critical-thinking disposition), we retained the validated multidimensional item sets and relied on the high composite reliabilities to support convergent validity (Fornell & Larcker, 1981; Malhotra & Dash, 2011), a deliberate choice that favors content validity over a numerical threshold. Both re-specifications that a stricter reading of AVE demands were tested in this revision (Appendix B). Stepwise removal of items loading below 0.60 lifts the technostress AVE above 0.50 only after discarding twelve of eighteen items, collapsing the scale onto its addiction facet, and leaves critical thinking at 0.446 even when no sub-0.60 item remains; the higher-order RED specification restores convergent validity at both levels and lowers the SRMR to 0.082, with structural conclusions unchanged throughout. These analyses bound the measurement concern: it affects the validity indices, not the findings. Modeling the critical-thinking disposition by facets, with the full inventory, remains a task for future instruments. AI self-efficacy is a recently introduced construct whose measurement continues to evolve, and future studies should triangulate it with AI-literacy and AI-attitude measures (Y.-Y. Wang & Chuang, 2024; B. Wang et al., 2022; Grassini, 2023; Carolus et al., 2023). The interaction effect was underpowered and should be re-examined with larger samples and complementary moderation techniques. Finally, the modestly explained variance points to unmeasured determinants of critical thinking; future models should incorporate instructional, dispositional, and motivational antecedents, employ objective critical-thinking assessments rather than self-report dispositions, and test the model across cultural contexts and academic disciplines (Kocak et al., 2021; Van et al., 2022).

## 5. Conclusions

This study examined whether technostress erodes critical thinking and whether there are more complex answers to this question than the dominant narratives suggest. In a sample of 340 university students in Peru, technostress showed no direct effect on critical thinking and did not serve as the pathway linking AI self-efficacy to critical thinking. What was of greatest importance was the participants’ self-efficacy in using AI. This was the only variable shown to have a strong positive relationship with critical thinking. The hypothesized moderation, under which that confidence would be most valuable at high technostress, was not supported under strict bootstrap inference; whether such a boundary condition exists is a question this study leaves open.

This study makes three distinct contributions. First, this study demonstrates the importance of domain-specific self-efficacy for social–cognitive theory in the context of AI use and describes technostress more accurately as a potential limiting condition rather than a direct threat. Second, and on a methodological note, this study combines traditional estimation and a comprehensive predictive battery with bootstrap inference and reports the limited applicability of the model outside the sample, rather than an overly optimistic estimate of the model’s scope. Third, at the practical level, this study proposes the development of AI skills and critical thinking skills within the context of AI literacy programs and the controlled use of AI as a more direct approach to fostering critical thinking in the Latin American context than stress reduction.

The conclusions drawn are limited by a cross-sectional, single-country approach and an early consideration of self-efficacy in AI as a construct. Therefore, the moderating role of technostress should be considered a hypothesis to be explored rather than a definitive finding. Future research should be longitudinal and span multiple contexts, include larger samples designed to detect interaction effects, and employ objective measures of critical thinking. That said, the main message holds: in AI-rich higher education, strengthening students’ confidence and competence with AI is likely a more effective lever for critical thinking than shielding them from technostress.

## Figures and Tables

**Table 1:** Sociodemographic characteristics of the sample (*n* = 340).

Characteristic	Category	*n*	%
Sex	Female	233	68.5
	Male	107	31.5
Age (years)	18–22	252	74.1
	23–27	46	13.5
	28 or older	42	12.4
University	Public	213	62.6
	Private	127	37.4
Year	First	98	28.8
	Third	136	40.0
	Other	106	31.2
Faculty	Health Sciences	144	42.4
	Humanities	107	31.5
	Business/Eng./Law	89	26.1
Employment	Not employed	203	59.7
	Employed/interning	137	40.3

**Table 2:** Descriptive statistics and normality of the study constructs.

Construct	M	SD	Skewness	Kurtosis	Response Scale
Critical thinking (CT)	3.66	0.52	−0.18	1.22	1–5
AI self-efficacy (SE)	3.23	0.72	−0.24	0.76	1–5
Technostress (TE)	2.19	1.01	0.74	1.13	0–6

Note. M and SD were computed on the mean score across each construct’s items; item-level skewness and kurtosis ranged within plus or minus 2, indicating no severe departures from normality.

**Table 3:** Measurement model: standardized loadings, bootstrap confidence intervals, reliability, and AVE.

Item	λ [95% CI]	t	α	ρA	ρc	AVE	VIF
**Critical Thinking (CT)**							
CT8	0.521 [0.422, 0.610]	10.8	0.878	0.884	0.898	0.405	1.46
CT10	0.579 [0.495, 0.652]	14.5					1.44
CT11	0.651 [0.591, 0.705]	22.6					1.53
CT12	0.650 [0.572, 0.721]	16.9					1.61
CT13	0.703 [0.638, 0.764]	21.8					1.89
CT15	0.605 [0.520, 0.683]	14.5					1.49
CT16	0.660 [0.587, 0.727]	18.3					1.70
CT17	0.665 [0.599, 0.723]	20.3					1.69
CT21	0.602 [0.525, 0.674]	15.7					1.49
CT22	0.692 [0.626, 0.748]	22.4					1.94
CT25	0.575 [0.489, 0.656]	13.5					1.41
CT26	0.642 [0.564, 0.711]	16.9					1.69
CT27	0.704 [0.636, 0.765]	21.4					1.81
**AI Self-Efficacy (SE)**							
SE1	0.698 [0.632, 0.758]	21.2	0.867	0.873	0.900	0.602	1.53
SE2	0.740 [0.668, 0.800]	22.2					1.75
SE3	0.806 [0.759, 0.847]	35.7					2.04
SE4	0.850 [0.808, 0.886]	42.4					2.45
SE5	0.786 [0.729, 0.833]	29.4					1.96
SE6	0.765 [0.709, 0.814]	27.6					1.91
**Technostress (TE)**							
TE3	0.539 [0.451, 0.618]	12.8	0.925	0.921	0.922	0.400	1.89
TE5	0.613 [0.533, 0.686]	15.6					2.45
TE6	0.646 [0.574, 0.710]	18.6					2.71
TE7	0.702 [0.641, 0.758]	24.1					3.28
TE8	0.640 [0.566, 0.703]	18.3					3.22
TE9	0.669 [0.606, 0.731]	21.2					3.09
TE10	0.561 [0.472, 0.640]	13.0					2.12
TE11	0.546 [0.454, 0.632]	11.8					2.50
TE12	0.631 [0.556, 0.698]	17.4					2.75
TE14	0.586 [0.499, 0.662]	13.9					3.06
TE15	0.558 [0.455, 0.644]	11.6					2.30
TE16	0.542 [0.444, 0.627]	11.4					2.12
TE17	0.669 [0.598, 0.729]	20.3					1.97
TE18	0.590 [0.510, 0.661]	15.4					2.15
TE19	0.641 [0.577, 0.701]	20.3					1.93
TE20	0.694 [0.633, 0.747]	23.9					2.37
TE21	0.776 [0.736, 0.813]	40.1					2.83
TE22	0.722 [0.663, 0.775]	25.3					1.93

Note. λ = standardized loading; 95% CI from 5000-resample bootstrap (fixed-weight, sign-stable); t = λ/bootstrap SE; α = Cronbach’s alpha; ρA = Dijkstra–Henseler rho; ρc = composite reliability; AVE = average variance extracted; VIF = indicator collinearity. Reliability indices are shown once per construct. Full item wording in Appendix A.

**Table 4:** Discriminant validity: Fornell–Larcker criterion and HTMT.

Construct	CT	SE	TE
CT	**0.636**	0.428	0.007
SE	0.407	**0.776**	0.192
TE	0.037	0.239	**0.632**

Note. Diagonal (bold) = square root of AVE. Below diagonal = inter-construct correlations (Fornell–Larcker). Above diagonal = HTMT ratios (threshold < 0.85; Henseler et al., 2015).

**Table 5:** Model fit indices.

Index	Value	Threshold	Decision
SRMR	0.105	<0.08 (strict); <0.10 (lenient)	Above 0.10 cutoff (limited exact fit)
NFI	0.952	≥0.90	Meets threshold
d_ULS	7.40	Bootstrap CI-based	Reported
d_G	7.25	Bootstrap CI-based	Reported

Note. Because the structural model is recursive and just-identified (saturated), the saturated and estimated models produce identical model-implied matrices, so d_ULS and d_G coincide across them. Following Hair et al. (2019), global fit is treated as secondary in PLS-SEM; inference rests on the measurement and predictive assessments.

**Table 6:** Explanatory power and predictive relevance.

Endogenous Construct	R^2^	R^2^adj	Q^2^predict (PLS < LM)
Technostress (TE)	0.057	0.054	0.009 (13/18)
Critical thinking (CT)	0.170	0.165	−0.003 (13/13)

Note. Q^2^predict from PLSpredict (k = 10 folds, 10 repetitions); “PLS < LM” = number of indicators for which PLS RMSE is below the linear-model benchmark.

**Table 7:** CVPAT: average loss difference vs. benchmarks.

Endogenous Construct	PLS—Indicator Avg.	PLS—Linear Model
Technostress (TE)	−0.016	−0.006
Critical thinking (CT)	+0.044	−0.744

Note. Negative values indicate lower prediction loss (better) than the benchmark. PLS surpasses both benchmarks for technostress (TE), but not the indicator-average benchmark for critical thinking (CT).

**Table 8:** Structural model and hypothesis testing (5000 bootstrap resamples).

Hyp.	Path	β	95% CI	t	*p*	f^2^	Decision
H1	TE → CT	−0.064	[−0.221, 0.114]	0.61	0.50	0.005	Not supported
H2	SE → CT	0.422	[0.324, 0.529]	8.15	<0.001	0.203	Supported
—	SE → TE	0.239	[0.122, 0.345]	4.17	<0.001	0.060	Robust †

Note. β = standardized path coefficient; CI = percentile bootstrap interval; f^2^: 0.02 small, 0.15 medium, 0.35 large (Cohen, 1988). Hypotheses judged on bootstrap CIs. † Standard (fixed-measurement) bootstrap interval; under full re-estimation of the measurement model in each resample this path is not robust (Section 3.5; Appendix B).

**Table 9:** Interaction effect and simple slopes.

Effect	Estimate	95% CI/Note
Interaction TE × SE → CT (two-stage)	0.146	[0.024, 0.251], *p* = 0.012
Interaction TE × SE → CT (full bootstrap)	0.146	[−0.015, 0.264], *p* = 0.07
Simple slope SE → CT at −1 SD technostress	0.305	low technostress
Simple slope SE → CT at mean technostress	0.451	
Simple slope SE → CT at +1 SD technostress	0.596	high technostress

**Table 10:** Mediation analysis: direct, indirect, and total effects (5000 bootstrap resamples).

Effect	Coefficient	95% CI	Decision
Path a: AI self-efficacy → TE	0.239	[0.122, 0.345]	Significant (*p* < 0.001)
Path b: TE → CT	−0.064	[−0.221, 0.114]	Not significant
Direct effect c′: AI self-efficacy → CT	0.422	[0.324, 0.529]	*p* < 0.001
Indirect effect a × b: AI self-efficacy → TE → CT	−0.014	[−0.039, 0.011]	Not supported
Total effect c: AI self-efficacy → CT	0.408	—	*p* < 0.001

Note. 95% CIs from percentile bootstrap. Zhao et al. (2010) classification = no effect (no mediation); VAF not interpretable because direct and indirect effects have opposite signs.

**Table 11:** Summary of hypothesis testing.

Hyp.	Path	β	Decision
H1	Technostress → Critical thinking	−0.06	Not supported
H2	AI self-efficacy → Critical thinking	0.42 ***	Supported
H3	Technostress × AI self-efficacy → Critical thinking	0.15 †	Not supported (significant only under two-stage; n.s. under full bootstrap)

Note. *** *p* < 0.001; † significant under the two-stage approach (*p* = 0.012) but marginal under the full bootstrap (*p* = 0.07).

**Table 12:** Multi-group analysis (public vs. private universities) with measurement invariance.

Path	Public β (*n* = 213)	Private β (*n* = 127)	Δ (Pub − Priv)	*p* (perm.)	Decision
AI self-efficacy → critical thinking	0.40	0.48	−0.09	0.43	No difference
Technostress → critical thinking	−0.05	0.02	−0.07	0.74	No difference
AI self-efficacy → technostress	0.28	0.32	−0.04	0.72	No difference
Technostress × AI self-efficacy → critical thinking	0.20	0.02	0.18	0.20	No difference

Note. Δ = difference between group path coefficients; *p* from a permutation test (Henseler et al., 2016); none significant at α = 0.05. MICOM established full measurement invariance (compositional invariance c = 0.88–0.99; equal composite means and variances, all *p* > 0.10).

## Data Availability

The data presented in this study are available upon reasonable request from the corresponding author.

## Appendix A. Item Wording (Faithful English Translations of the Administered Spanish Items)

**Table A1:** The full wording (English translation) of the retained items.

Code	Item
**Critical Thinking (CT)**	
CT8	I explain my reasons when I disagree with others
CT10	Even when something is already proven, I still ask questions about it
CT11	I have a reputation for being a rational person
CT12	I continually evaluate whether my thinking is correct before forming an opinion
CT13	I continually seek information related to solving a problem
CT15	I willingly solve a complicated problem
CT16	When I look at the world, I do so with an inquiring mind
CT17	I believe I can solve any complicated problem
CT21	I try to understand how the unknown works
CT22	When facing a problem, I strive to find an answer until I solve it
CT25	When solving a problem, I organize data systematically
CT26	I fairly evaluate both my own opinion and that of others
CT27	I trust my own judgment to solve problems
**AI Self-Efficacy (SE)**	
SE1	If someone opposes me, I can find ways to get what I want using AI
SE2	It is easy to stay true to my goals and accomplish them with AI
SE3	I am confident I could deal efficiently with unexpected events using AI
SE4	Thanks to my resourcefulness supported by AI, I handle unforeseen situations
SE5	I stay calm facing difficulties because I trust my AI-supported coping
SE6	No matter what comes my way, I can usually handle it with AI support
**Technostress (TE)**	
TE3	I distrust whether technologies contribute anything to my studies (skepticism)
TE5	I find it hard to relax after a day of studying using them (fatigue)
TE6	When I finish studying with ICT, I feel exhausted (fatigue)
TE7	I am so tired after studying with them that I cannot do anything else (fatigue)
TE8	It is hard to concentrate after studying with technology (fatigue)
TE9	I feel tense and anxious when studying with technology (anxiety)
TE10	It frightens me that I could destroy information through improper use (anxiety)
TE11	I hesitate to use technology for fear of making mistakes (anxiety)
TE12	Studying with them makes me uncomfortable, irritable, impatient (anxiety)
TE14	It is difficult to study with ICT (inefficacy)
TE15	People say I am ineffective at using technologies (inefficacy)
TE16	I am unsure about finishing my tasks well when I use ICT (inefficacy)
TE17	I think I use technology excessively in my life (addiction)
TE18	I continually use technology, even outside study hours (addiction)
TE19	I think about technologies continually, even outside study hours (addiction)
TE20	I feel anxious if I do not have access to technology (addiction)
TE21	An inner urge compels me to use them anywhere, anytime (addiction)
TE22	I devote more time to technology than to friends, family, and hobbies (addiction)

## Appendix B. Robustness and Re-Specification Analyses

The analyses in this appendix were added during the revision process. They use the study dataset (*n* = 340) and an independent implementation of the PLS-PM algorithm (mode A outer estimation, path weighting scheme). Before any re-specification was run, the implementation was verified against the published model: it reproduces every loading, AVE, reliability coefficient, HTMT ratio, path coefficient, R^2^, and the SRMR reported in Section 3.1, Section 3.2, Section 3.3, Section 3.4 and Section 3.5 to the third decimal. Confidence intervals below come from 5000-resample bootstrapping with full re-estimation of the measurement model in each resample, the conservative variant of Section 3.6; this is why some intervals are wider than their fixed-measurement counterparts in Table 8 (for the AI self-efficacy → technostress path, fixed-measurement resampling reproduces the interval reported there). The analysis code is available from the corresponding author.

Table A2 applies the stepwise purification procedure suggested during the review process: at each step the item with the lowest standardized loading below 0.60 is removed and the full model is re-estimated. Two results stand out. First, the technostress AVE passes 0.50 only at the thirteenth step, by which point the scale has lost its skepticism, fatigue, anxiety, and inefficacy content entirely and has collapsed onto the six addiction items. Second, the procedure terminates for critical thinking at AVE = 0.446: once no item loads below 0.60, further trimming is not licensed by the rule itself, so the 0.50 benchmark cannot be reached for this disposition without discarding items the criterion says to keep. Throughout the sixteen models the structural estimates barely move (AI self-efficacy → critical thinking between 0.42 and 0.44; technostress → critical thinking between −0.06 and −0.08, never significant), and the SRMR improves from 0.105 to 0.067. Measurement heterogeneity therefore affects the validity indices, not the substantive conclusions.

**Table A2:** Stepwise item purification (removal of the lowest loading below 0.60, one item at a time).

Step	Item Removed (λ)	AVE CT	AVE TE	β SE → CT	β TE → CT	SRMR
0	— (baseline)	0.405	0.400	0.422	−0.064	0.105
1	CT8 (0.521)	0.417	0.400	0.427	−0.065	0.107
2	TE3 (0.539)	0.417	0.402	0.430	−0.077	0.108
3	TE11 (0.525)	0.417	0.406	0.430	−0.077	0.104
4	TE16 (0.522)	0.417	0.413	0.430	−0.076	0.102
5	TE15 (0.532)	0.417	0.420	0.430	−0.072	0.099
6	TE10 (0.525)	0.417	0.429	0.430	−0.072	0.097
7	TE14 (0.531)	0.417	0.438	0.429	−0.068	0.093
8	TE5 (0.564)	0.416	0.444	0.430	−0.071	0.090
9	TE12 (0.540)	0.416	0.457	0.430	−0.072	0.086
10	TE8 (0.545)	0.416	0.474	0.431	−0.072	0.078
11	TE6 (0.552)	0.416	0.498	0.431	−0.074	0.072
12	TE9 (0.503)	0.416	0.535	0.432	−0.075	0.067
13	TE7 (0.529)	0.416	0.586	0.431	−0.071	0.067
14	CT10 (0.577)	0.427	0.586	0.436	−0.082	0.068
15	CT25 (0.586)	0.446	0.586	0.416	−0.081	0.067

Note. Each row describes the re-estimated model after that removal; β from the main structural model, 5000-resample full bootstrap available for the final model (β SE → CT 95% CI [0.321, 0.529]; β TE → CT 95% CI [−0.199, 0.041]). The procedure stops when no loading falls below 0.60. Items TE17–TE22 form the addiction facet of the RED scale.

Table A3 reports the specification recommended in review as the alternative to trimming: the five RED facets modeled as lower-order reflective constructs beneath a global technostress factor (disjoint two-stage approach). Convergent validity is restored at both levels. Facet AVEs range from 0.586 (addiction) to 0.759 (inefficacy), with composite reliabilities of 0.895 to 0.912, and the higher-order construct shows AVE = 0.568 with facet loadings between 0.689 and 0.807. The structural story is unchanged: AI self-efficacy predicts critical thinking (β = 0.417, 95% CI [0.321, 0.521]), technostress does not (β = −0.043, 95% CI [−0.215, 0.132]), R^2^ for critical thinking is 0.168, and the SRMR falls to 0.082. The below-threshold AVE of the single-factor operationalization therefore reflected facet heterogeneity, not measurement error.

**Table A3:** Higher-order re-specification of technostress: measurement quality at both levels (disjoint two-stage approach).

Construct	Items	λ Range	AVE	ρc
Skepticism (SKE)	1 (TE3)	single indicator	—	—
Fatigue (FAT)	4 (TE5–TE8)	0.754–0.908	0.723	0.912
Anxiety (ANX)	4 (TE9–TE12)	0.788–0.889	0.711	0.908
Inefficacy (INE)	3 (TE14–TE16)	0.802–0.919	0.759	0.904
Addiction (ADD)	6 (TE17–TE22)	0.689–0.824	0.586	0.895
Technostress (higher order)	5 facet scores	0.689–0.807	0.568	0.867

Note. First-stage facet scores serve as indicators of the higher-order construct in the second stage. Skepticism retains a single item after the a priori exclusions described in Section 2.2 and enters as a single-indicator construct. Structural results (full bootstrap): SE → CT β = 0.417 [0.321, 0.521]; TE → CT β = −0.043 [−0.215, 0.132]; SE → TE β = 0.224 [−0.235, 0.357]; R^2^(CT) = 0.168; SRMR = 0.082.

The interaction term was re-examined under both re-specifications. Under the higher-order model it stays non-significant with the full bootstrap (β = 0.142, 95% CI [−0.032, 0.254], *p* = 0.099), while the two-stage variant is significant (95% CI [0.029, 0.244]), the same divergence reported in Section 3.6. Under the trimmed, addiction-focused model the interaction is significant even under full re-estimation (β = 0.146, 95% CI [0.021, 0.280], *p* = 0.024). The conditional effect therefore depends on how technostress is operationalized: it emerges when the scale narrows to compulsive-use content and dissolves under the theory-faithful facet structure. We read this specification-dependence as confirming the decision to reject H3, and as marking facet-level moderation, addiction in particular, as the precise question for future, adequately powered designs.

Table A4 implements the predictive benchmark suggested in review. Latent variable scores from the higher-order measurement model were extracted within each training partition (outer weights, means, and variances estimated on training folds only, so no test information enters the measurement model) and passed to ordinary least squares, ridge regression with the penalty tuned by inner five-fold cross-validation, and support vector regression with a radial kernel, under ten repetitions of 10-fold cross-validation. All three learners beat the training-mean benchmark at the construct-score level, and none beats ordinary least squares. Regularization and nonlinearity add nothing here, which locates the modest predictive ceiling in the constructs measured rather than in the estimator and is consistent with the indicator-level PLSpredict results of Section 3.4.

**Table A4:** Out-of-sample benchmark at the construct level: ten repetitions of 10-fold cross-validation.

Model	RMSE	R^2^ Out-of-Sample
Training-mean benchmark	1.006	0.000
Ordinary least squares	0.924	0.156
Ridge regression (CV-tuned penalty)	0.925	0.154
Support vector regression (RBF kernel)	0.938	0.131

Note. Target = critical-thinking latent score; predictors = AI self-efficacy, technostress (higher order), and their product. RMSE on standardized scores, pooled over 100 test folds.
